# Design of a soft robotic endoscope with enhanced bending and AI-based prediction

**DOI:** 10.1038/s41598-026-46334-y

**Published:** 2026-04-20

**Authors:** Miriam Hani, Mohamed N. Elghitany, Rania Sweif, Shady A. Maged

**Affiliations:** 1 Mechatronics Engineering Department, Faculty of Engineering, Ain Shams University, Cairo, 11566 Egypt; 2 Electrical Power Engineering Department, Faculty of Engineering, Ain Shams University, Cairo, 11566 Egypt

**Keywords:** Bending angle, Endoscope, Finite element analysis, Surgical applications, Artificial neural networks, Support vector machine, Engineering, Mathematics and computing

## Abstract

Minimally invasive surgery (MIS) increasingly benefits from soft robotic platforms that offer enhanced compliance and safer human–robot interaction. However, one of the most important challenges of design is still to achieve larger bending at low actuation pressure while maintaining structural integrity. This paper introduces a proposed novel soft robotic endoscope aimed at maximizing bending performance and reducing needed pressure in complex surgeries below the maximum limit of blood pressure, which is 0.24 bar (Boutouyrie in Hypertension 34(4):475–480, 1999), to enhance patients’ safety and reduce their trauma. The proposed design demonstrates significant improvements in both bending angle and operating pressure compared to existing architecture. Finite element modeling was utilized to evaluate the performance of the novel design under several test cases. Then, machine learning techniques were implemented to develop an Artificial Intelligence (AI) model aimed at predicting the most effective parameters needed for testing the design. Instead of implementing Finite Element Analysis for each new design, a highly accurate model will now predict bending angle, twisting angle, bending speed and twisting speed for the proposed design under any input pressure conditions. Experimental validation was developed by fabricating the module and testing it to validate the proposed design’s performance. The results show that the proposed design achieves higher bending angles at same pressures, compared to previous designs, under the specified design conditions.

**Supplementary Information:**

The online version contains supplementary material available at 10.1038/s41598-026-46334-y.

## Introduction

Over the past two decades, the surgical landscape has been undergoing a quiet but powerful transformation. With increasing pressure to reduce patient trauma, recovery times, and hospital stays, Minimally Invasive Surgery (MIS) has emerged as the preferred alternative to conventional open surgery. Procedures that once required large incisions and extensive recovery can now be performed through a few small ports, aided by advanced tools such as endoscopes. Originally designed as passive visual aids, endoscopes have rapidly evolved into multifunctional platforms that enable manipulation, dissection, and even localized therapy^2,3^.

Yet, despite these advancements, traditional endoscopic systems still face some challenges. Rigid endoscopes offer stability and force transmission but struggle with anatomical complexity, especially when reaching behind organs or around delicate structures. Flexible endoscopes provide better access but lack the stiffness control required for accurate manipulation and are more likely to loop or buckle in convoluted pathways. These limitations have driven the search for a new class of surgical instruments, which are soft robotic endoscopes, that combine flexibility with functionality^2–4^.

Soft robotics introduces a fundamentally different approach. Inspired by biological structures like octopus’ arms and elephant trunks, these devices are constructed from compliant, biocompatible materials and actuated using pneumatic, hydraulic, or smart-material-based mechanisms. Their inherent softness allows safer navigation through the body, adapting to anatomical curves while reducing the risk of tissue damage. Moreover, recent advances in variable stiffness technologies, such as granular jamming, layer jamming, and shape memory materials, allow these systems to transition between flexible and rigid states during procedures, making them uniquely suited to the dynamic demands of MIS and NOTES (Natural Orifice Transluminal Endoscopic Surgery)^5–8^.

Recent advancements in soft robotic endoscopy have introduced a variety of design strategies, offering novel solutions in actuation mechanisms, module flexibility, and potential for use in complex surgical applications. Gifari et al.^9^ introduced the STIFF-FLOP manipulator, which passed by many improvements. The manipulator’s first design offered flexibility but suffered from friction. The second improved bending but lost torque. The third increased strength and stability, yet friction remained. Overall, each version balanced benefits and drawbacks, showing the challenge of achieving both flexibility and efficiency in soft robotics for MIS^8,9^.

Naghibi et al.^10^ proposed a design with three-chamber model which achieved larger bending than STIFF-FLOP design, where the length of the module is 40 mm and the diameter is 28 mm. By applying a pressure of 0.3 bar to one chamber, the model could bend with 20°. Another proposed design with the same dimensions is four-chamber module, which bends at the same pressure with a higher angle which is 30°.

Decroly et al.^11^ developed a soft pneumatic actuator capable of two degrees of freedom for endoscopic navigation. This model was a two degree of freedom pneumatic actuator designed to bend in multiple directions, and it is not specific for medical applications. It has a diameter of 6 mm and demonstrates bending over 180° at low forces. However, this design has some drawbacks mentioned by the authors, particularly manufacturing caused imperfections that lead to deviations in the expected bending direction, resulting in unpredictable and non-ideal displacement behavior. The requirement of sophisticated modeling is also another issue as it is required to predict performance with high accuracy as the present model may not fully statistical the non-linear behavior of the soft actuator under different conditions.

Lenssen et al.^6^ implemented a four-chamber design achieved the highest bending angle of 117° at a relatively low input pressure of 0.6 bar. The external sheathing is used to limit radial expansion and enhancing longitudinal deformation, therefore increasing actuation efficiency. However, the design still faces several practical challenges. For one, achieving enough bending often requires higher air pressure, which could raise safety concerns during actual surgical use. While some chamber shapes help make the device more compact, they can also reduce how well it bends or make the system harder to manufacture, making it difficult to miniaturize the device, which is essential for minimally invasive procedures.

Elghitany et al.^12^ introduced a novel soft robotic endoscope based on a Parallel Rings Design (PRD), which attains a maximum bending angle of approximately 82.8° at a pressure of just 0.2 bar, with improved stiffness modulation via parallel rings. A lack of embedded sensing devices for real-time feedback, which is essential for surgical applications, and fine-tuned stiffness control by using multi-pressure actuation is still difficult. Additionally, further miniaturization is necessary to meet the spatial limitations of minimally invasive surgical tools.

MIS continues to drive demand for surgical tools that are not only smaller and more flexible but also safer and easier to control. Traditional endoscopes, while widely used, often fail to adapt to the intricate and curved paths of human anatomy, increasing the risk of tissue damage or procedural limitations^13^. Table 1 shows an overview of previous endoscopic designs.

In this paper, a novel soft pneumatic module is presented which addresses these challenges through a redesigned actuation architecture aimed at reducing input pressure while enhancing bending performance. This innovative design not only minimizes the risk of tissue damage but also allows for greater flexibility in navigating complex anatomical structures.

## Principle of design

To find the best design that could solve the challenges found in previous soft endoscopes, many different design ideas were investigated. The first concept was not immediately implemented, but several modified designs were developed, each with small changes to improve how well the endoscope could bend, how much pressure it needs, and how easy it would be to build. For every design, the behavior is studied carefully, and the results are compared. The following section shows our proposed novel endoscope design which is improving system’s performance.

**Fig. 1:**
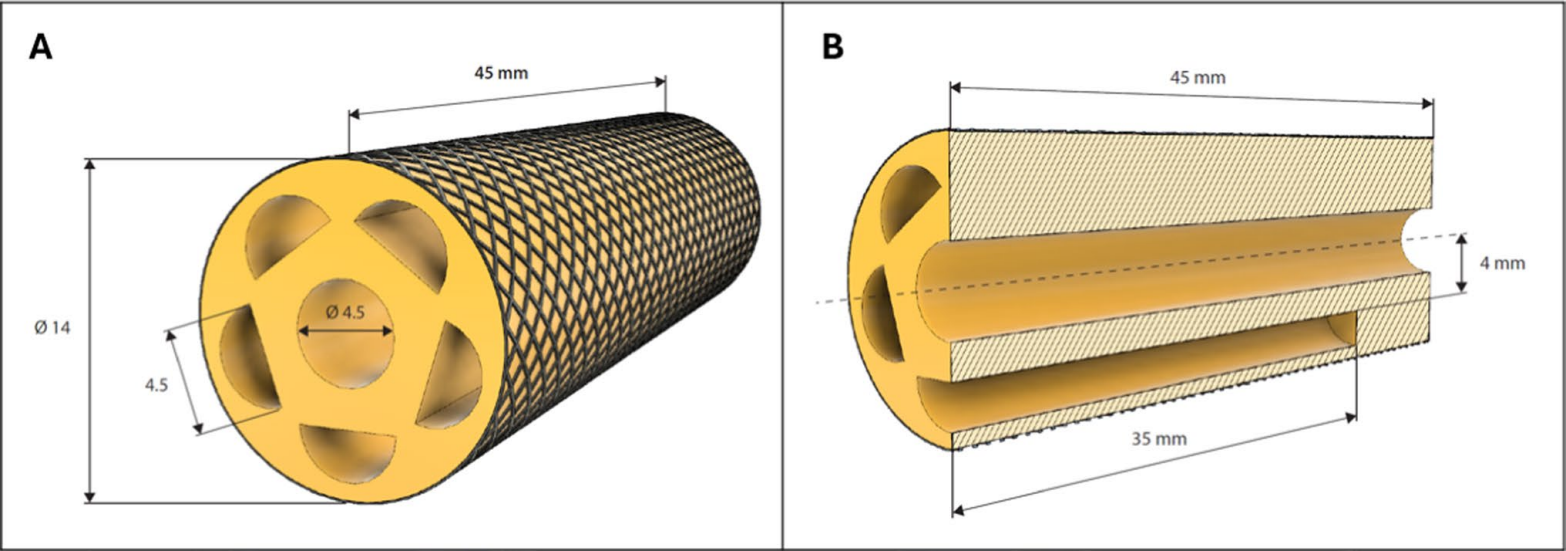
Schematic Representation of proposed endoscope. (**A**) Proposed Module 3D design, (**B**) Lateral section of the proposed mold.

Many of the drawbacks of earlier systems are effectively addressed by the suggested design. Among these restrictions were the use of external sheaths, which created friction and resulted in energy losses during actuation, and inadequate bending angles. Furthermore, prior designs frequently depended on constrained testing conditions, which made it challenging to forecast performance in a variety of real-world situations. To get around these problems, the system was tested and simulated under different loading scenarios using finite element analysis (FEA), which gave a more thorough understanding of how the system behaved. With this method, the design can achieve higher bending angles, with a reduced sensitivity to pressure fluctuations and material non-linearities, making it a reliable solution under different operating conditions. Our novel endoscope design is cylindrical in shape with 5 chambers distributed among 360° with semicircular shape. Semicircular shapes are chosen in chambers’ design as they are asymmetrical shapes, generating a higher bending moment compared to circular symmetrical shapes. This results in reducing radial expansion, which lowers external contact area for the same applied pressure. The number of chambers has increased to 5 as increasing the number of chambers has proved to have a direct relation in increasing the bending angle, Naghibi et al.^10^ Thus, fewer chambers usage reduces directional bending and requires simultaneous actuation of adjacent chambers^7^. While increasing the number of chambers has a benefit in increasing the resulting bending angle, but it reduces chambers size which leads to high fabrication sensitivity; leading to increasing control complexity and make it harder to fabricate and test the module^7^. Our endoscope is covered with fiber outer sheath to maintain system’s stability and avoid ballooning effect that can occur when pressure is applied and ensures that the endoscope retains its shape even under varying internal pressures. Ballooning effect is the outward radial expansion of the module which reduces bending efficiency and may cause exposures in human body with higher pressures. The sheath is designed to be lightweight and flexible, allowing the endoscope to bend and deform as needed without adding significant stiffness and without affecting initial endoscope’s dimensions. Importantly, the sheath does not exert a large force on the internal structure of the endoscope; instead, it acts primarily as a stabilizing element. This ensures that while the internal pneumatic chambers are pressurized to achieve bending, the sheath prevents excessive outward expansion and helps control the uniformity of deformation^9^. Consequently, our design attains an elevated bending angle, achieving 90°, which exceeds the performance established by Elghitany et al.^12^. Actuating different chambers with different pressures will directly affect bending angles, twisting angles and surely the direction of motion. To further confirm the design’s effectiveness, we conducted FEA, where we tested the endoscope under a range of conditions, we tried to test most of the expected and possible conditions. This testing helps overcome the limitations highlighted by Decroly et al.^11^, especially regarding material behavior and how the endoscope performs under varying pressure conditions. Because of this, our design is the best choice for surgical applications where greater flexibility and dependability are desired. The detailed design is shown in Fig. 1 with all the dimensions in mm.

## Finite element analysis

**Fig. 2:**
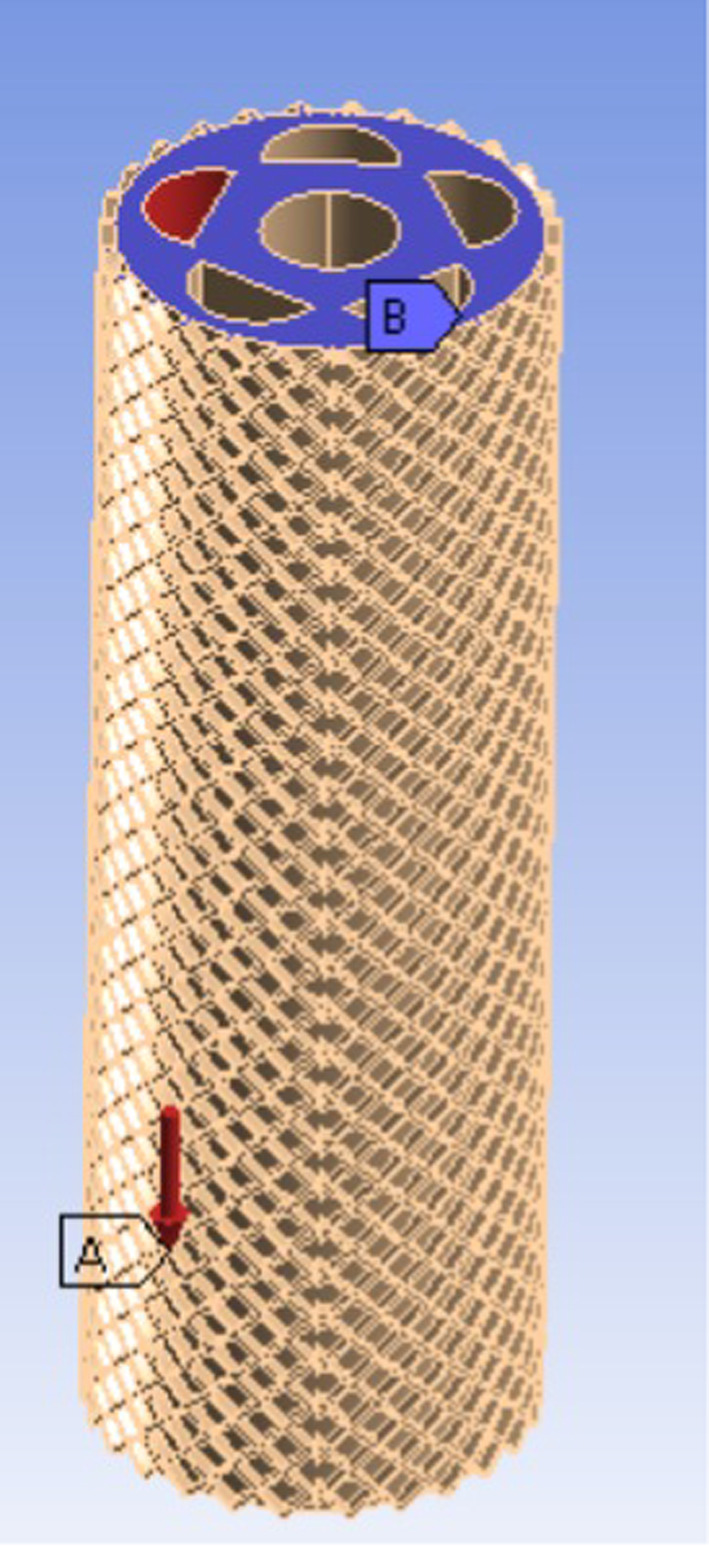
Finite Element Analysis.

FEA, Fig. 2, is necessary for soft robots’ endoscopes as it allows accurate modeling and simulation of their complex, deformable behavior, which is difficult to achieve with conventional analytical methods. FEA is a valuable tool in the design process because it allows us to test and improve soft robots virtually before building them. By simulating how the structure behaves under different loads and conditions, weaknesses can be spot, the design can be adjusted, and reliability can be tested; all without the cost and time of repeated physical prototypes. Finite element simulations were performed using ANSYS Workbench (v2020R2) to evaluate the structural performance of the proposed soft robotic endoscope under internal pneumatic actuation. The Static Structural module was selected to analyze the deformation response under steady-state pressure conditions, which is appropriate for the quasi-static nature of soft robot actuation. A hyperelastic material model was defined using the Yeoh formulation, suitable for capturing the large, nonlinear deformations typical in soft silicone-based materials. Material parameters were assigned to reflect the properties of Ecoflex™ 00–50, a widely used elastomer in soft robotics. Elastomer Sample’s properties are set according to datasheet of Ecoflex™ 00–50 with density of 1070 kg/m^3^. Yeoh second order model is used to simulate the system, which energy function is:

1 $$W = C_{10} \left( {I_{1} - \, 3} \right) + C_{20} \left( {I_{1} - \, 3} \right)^{2}$$

where I_1_ is the first invariant of deformation.

Material parameters used are C_10_ = 0.045 MPa, C_20_ = 0.01 MPa^13,14^. The geometry is imported as step file, then opened in Design Modeler where we specify the material for each part of the body: the endoscope body is made of Ecoflex and the outer sheath is made of fiber. Selecting the appropriate mesh size is one of the most critical and challenging aspects of the simulation process. After conducting several surveys, research studies, and trials to determine the most suitable mesh size for our application, we selected a mesh size of 1 mm, according to mesh quality study. We conducted a mesh quality study using element sizes of 2 mm, 1.5 mm, 1 mm and 0.7 mm. The bending angle was observed, and less than 2% variation was observed between the 1 mm and 0.7 mm meshes, indicating convergence. Mesh quality was evaluated using skewness metrices, with average values below 0.2 and maximum values below 0.45, which is a good mesh quality.

In all tests, the “Large Deformation” option is activated to take into consideration the significant deflections produced by the soft surfaces^12,16,17^.

## Design parameters and mathematical model

This section introduces a detailed mathematical model of a soft robotic endoscope actuated by five internal chambers with semicircular shape, describing the kinematic behavior and dynamic response. They exhibit complex nonlinear behaviors due to their hyperelastic material properties, pressure-induced deformation, and geometric nonlinearity^15^. The model captures both bending and twisting dynamics. The model includes pressure input effects, damping and elastic restoring forces, Table 1.

**Table 1:** Overview of previous pneumatic actuated soft endoscope modules.

Author	Research	Drawback
Gifari et al.^9^	STIFF-FLOP design	Frictional losses due to external sheath
Naghibi et al.^10^	Three-chamber and four-chamber modules with semi- circular shape	The bending angle achieved is only 20° at 0.3 bar and 30° at the same pressure for the four-chamber module
Decroly et al.^11^	Two degrees of freedom pneumatic actuator that can achieve 180° at low forces	It is not functional at high forces and performance is unpredictable which is not safe for surgical applications
Lenssen et al.^6^	Comparing several designs while varying the number of chambers, chambers designs, and sheath used	Issue in achieving different bending angles
Elghitany et al. (2023)	PRD	Achieving 82.8° at pressure 0.2 bar, but varying bending angle and stiffness is not applicable

### Geometry

**Fig. 3:**
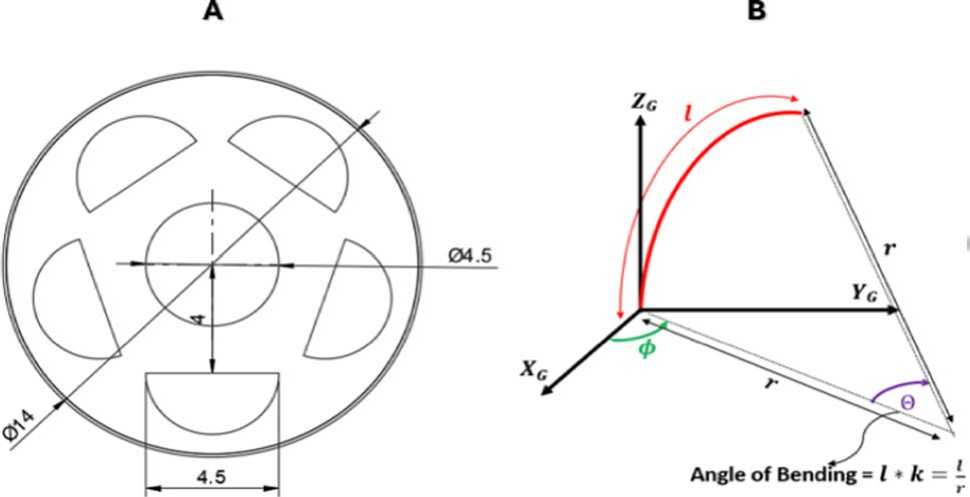
(**A**) Top View of the proposed endoscopic model, (**B**) Piece-Wise Constant Curvature Model.

The module is a cylindrical elastomer with five circumferentially equally spaced internal chambers. Each chamber can be independently actuated or in combination with any other one. The dimensions of the module, Fig. 3A, are minimized more than the previous modules. This new soft robotic endoscope is made smaller and more flexible than earlier designs, so it can fit easily into tighter spaces inside the body.

The following mathematical formulation will construct the system’s model to predict and control the bending, twisting, and overall behavior of the soft robotic endoscope under varying internal chamber pressures^18^. The geometry, material properties, and chamber positions influence the resulting motion^8^. Table 2 shows the detailed dimensions used in mathematical model’s implementation.

**Table 2:** Geometric Parameters for the proposed novel design.

Parameter	Value
Second Moment of Inertia I	493 mm^4^
Total Length L	45 mm
Segment Length L_n_	45 mm
Number of segments N	1
Chamber Cross Sectional Area A	100.5 mm^2^
Arc length d	14 mm
Chambers Angular Positions	0°, 72°, 144°, 216°, 288°

Ecoflex00-50 is used in our model due to its high flexibility and ability to undergo large deformations without damage.

It ensures safe interaction with internal tissues during surgeries which reduces the risk of injury^19^. Table 3 shows the material properties of Silicone^20^.

**Table 3:** Material properties of Silicone^14^.

Parameter	Value
Density ρ	1070 kg/m^3^
Young’s Modulus E	1.25 bar
Poisson’s Ratio ν	0.49
Shear Modulus μ	0.417 bar

### Kinematics

The soft robotic endoscope achieves its motion through the independent pressurization of multiple internal chambers that are symmetrically positioned along its body. To capture its deformation behavior with sufficient accuracy, the continuum structure is represented using the piecewise constant curvature (PCC) approach, which divides the body into N segments. Each segment bends independently due to localized internal pressurization^9,10,21^, Fig. 3B, where ø is the twisting angle, $\theta$ is the bending angle, l is the segment’s length and k is the curvature.

Five internal chambers, spaced at equal 72° angular intervals, are symmetrically arranged around the central neutral axis in each segment. The pressure inside each chamber is independently controlled to produce a differential stress distribution and a controllable bending moment.

Each segment is controlled by five pressure inputs, corresponding to the five chambers: Dorsal, Torsion, Lateral Right, Lateral Left and Ventral. The average axial pressure is:

2 $$Fp = \sum\nolimits_{(i = 1)}^{5} {P_{{i^{*} }} } \pi^{*} (D/2)^{2}$$

where P_i_ is the corresponding pressure on each chamber and D is the module’s diameter.

The local bending curvature vector for the n^th^ segment is denoted by,^20^:

3 $$Kn = \frac{Ad}{{EI}}\mathop \sum \limits_{i = 1}^{5} P_{n} \left[ {\begin{array}{*{20}c} {{\mathrm{Cos}} \theta n} \\ {{\mathrm{Sin}} \theta n} \\ \end{array} } \right]$$

where $\theta n \text{is the angular position of the chamber}$,I is the second moment of inertia; A, d and E are specified in Tables 2 and 3.

Thus, the bending angle can be calculated using the following Eq. (8),^18^:

4 $$\theta = {\mathrm{r}}_{{\mathrm{n}}} *{\mathrm{L}}_{{\mathrm{n}}}$$

where r_n_ is the radius of bending and L_n_ is the segment’s length.

### Dynamics

In addition to static bending, the soft robotic endoscope must also handle the dynamic effects that come from its flexible material and the way it is pressurized. When certain chambers are inflated more than others, they create bending forces that curve each segment in a controlled direction. At the same time, uneven pressure patterns can cause twisting around the central axis, which the soft silicone body naturally resists through its elasticity. This means that as the internal pressures change, the material’s bending stiffness and resistance to twisting work together to maintain the shape and stability of the device^7,21,22^.

Silicone material exhibits nonlinear behavior. We use a hyperelastic restoring force model^21^

5 $${\mathrm{F}}_{{\mathrm{e}}} = {\text{ E}}.{\text{ tanh }}\left( {\frac{Li - L0}{{L0}}} \right)$$

where E is Young’s modulus shown in Table 3, L_i_ is the final segment’s length after deformation and L_0_ is the initial segment’s length.

## Model analysis

In this section, we introduce the AI-driven, data-based simulation model we developed to predict how our soft robotic endoscope deforms in response to different internal pressures on chambers. The goal was to estimate the resulting bending angles when various pressures are applied to the endoscope’s chambers. This approach helps in consuming time during the design process and reducing the need for trial-and-error experimentation. To build this model, we followed multiple steps. First, we created a Python simulation environment, complete with a graphical user interface (GUI), so we could easily visualize how the robot would behave under different pressure scenarios. Next, we generated a dataset of 100 samples, each one representing how the actuator responded to a specific pressure configuration. Finally, we trained machine learning models using Artificial Neural Networks (ANNs) and Support Vector Machines (SVMs) to use this data and accurately predict the actuator’s behavior.

### Data collection and model analysis

As part of the design process for this soft robotic endoscope, intended for use in MIS, a Python-based simulation environment was created to predict how the device would perform under different conditions. This tool implements the full mathematical model, combining the chamber geometry, material elasticity, and bending and twisting mechanics into an interactive framework. All the important design parameters are included in the simulation, such as the size and configuration of the internal chambers, the Ecoflex 00–50 material’s elastic qualities, and the governing equations that explain the deformation behavior under various internal pressures. A user-friendly GUI was created so that the user could instantly view the bending curvature, bending angles, and twisting angle, if present, while adjusting individual chamber pressures or applying pressures simultaneously to several chambers. The specific simulation conditions and parameters used in this study are described in the following section.

In this model, the device is represented as a chain of 1 segment, each defined by an initial length of 45 mm and a diameter of 15 mm. Each segment contains five internal chambers symmetrically distributed around the neutral axis. All chambers are initialized with zero internal pressure and can be pressurized independently to generate controlled bending and twisting along the body. Bending and twisting are limited by practical maximums set in the model to prevent unrealistic deformation: the bending angle for each segment is capped at 90°, while the twisting angle is limited to 45°. To ensure physical feasibility, the maximum allowable bending moment per segment is defined as 0.005 Nm, and the maximum twisting torque is limited to 0.003 Nm^18^.

The interaction between the soft robotic endoscope and its environment is modeled by considering two main external effects: tissue contact at the distal tip and the continuous constraint provided by an external protective sheath surrounding the soft structure. When the simulated tip approaches or reaches a defined virtual boundary representing anatomical tissue, a restoring contact force is applied to prevent unrealistic penetration and to mimic the resistance encountered during real procedures^21^. This axial contact force is expressed as:

6 $${\mathrm{F}}_{{{\mathrm{contact}}}} = {\mathrm{K}}_{{{\mathrm{contact}}}} \left( {{\mathrm{z}}_{{{\mathrm{threshold}}}} - {\mathrm{z}}_{{{\mathrm{tip}}}} } \right)$$

where F_contact_ is the force applied on the external surface of endoscope, K_contact_ is the stiffness constant, z_threshold_ is the deformation threshold and z_tip_ is the actual deformation measured from the tip of endoscope^21,22^.

The resulting reaction torque that opposes further bending and twisting at the contact point is defined by:

7 $$\tau_{{{\mathrm{contact}}}} = - \alpha *{\mathrm{F}}_{{{\mathrm{contact}}}}$$

where α represents the distance from the force to the point of rotation and F_contact_ is the contact force applied on the module.

**Fig. 4:**
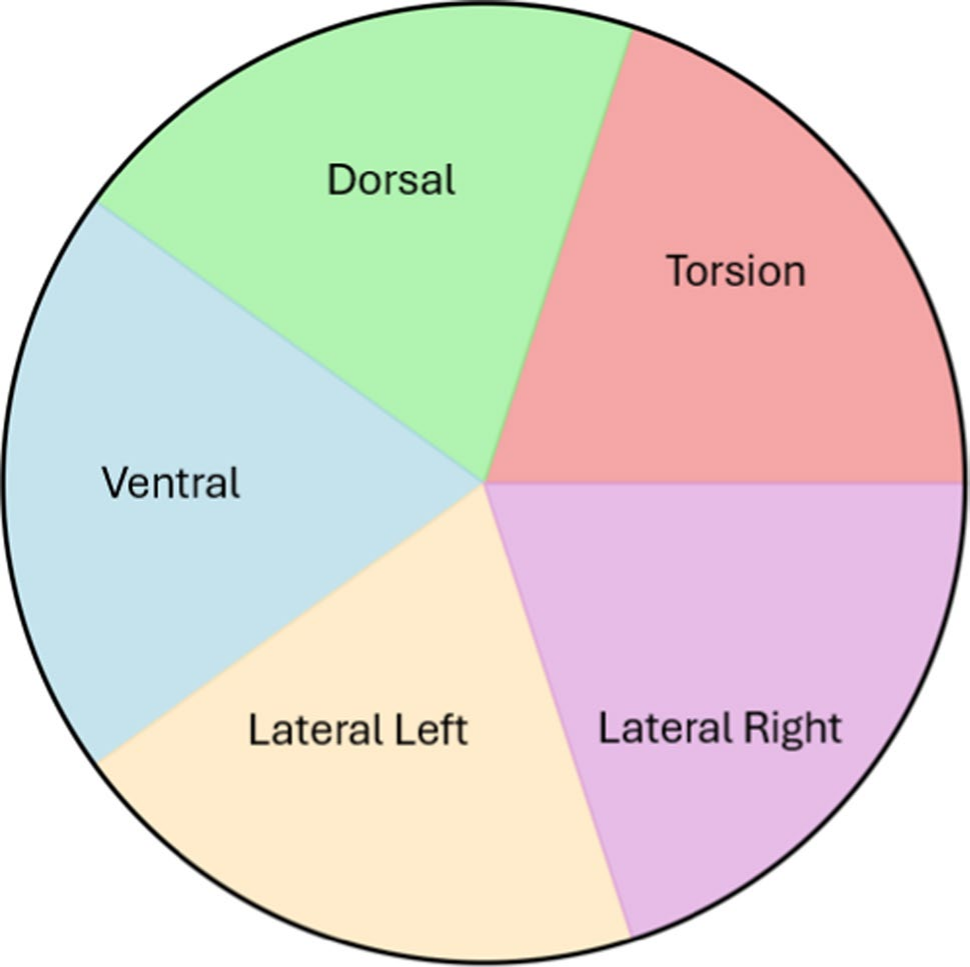
Chambers distribution with pressures applied.

The chambers are modeled as the following: Dorsal, Torsion, Lateral Right, Lateral Left and Ventral chamber, Fig. 4. These names describe the location of the chamber and the bending direction during actuation. Torsion chamber describes the twisting along the longitudinal axis of endoscope. Dorsal and Ventral are generally used to describe the motion toward the back and front of the body. The pressure applied to Lateral Left and Lateral Right chambers generate controlled side-to-side bending. Table 4 shows five different test cases, while linearly increasing ramp input pressures are applied with different values. In test case 1, a pressure of 0.2 bar is applied on Torsion and Ventral chambers; it results in a twisting angle of 118° and bending angle of 77°. The results in case 1 demonstrate that, even when the bending angle is large, its effect fades. This is because a large twisting angle directly influences bending behavior. To preserve the torsional shape, some of the internal stress is redirected as the structure twists. This can change the bending plane or marginally decrease the effective bending curvature. In test case 2, a pressure of 0.2 bar and 0.1 bar are applied respectively on Dorsal and Right Lateral chambers. The resulting bending angle in case 2 is 72° and the twisting angle is 0°. The absence of twisting angle in that case makes the module bend with the angle exactly and without any losses. In case 3, we tried the same values of pressures of case 2 but with changing chambers, a pressure of 0.2 bar and 0.1 bar are applied respectively on Right Lateral and Dorsal chambers. The bending and twisting angles in case 3 are approximately the same as case 2 but in opposite direction; and this is how to change bending direction for our module. In case 4, we actuated three chambers and not only two, pressures of 0.2, 0.1 and 0.1 bar were applied respectively on Dorsal, Right Lateral and Left Lateral chambers. The bending angle is noticed to be 59.16° with no twisting angle. In case 5, we actuated all the five chambers together while applying a pressure of 0.2 bar for each chamber. In this case, no bending angle occurs due to absence of differential strain caused by symmetrical deformation of the module. However, twisting angle is 87.43°; which is undesirable in endoscopic applications. Higher twisting angles may reduce controlling direction and accuracy. These angles are obtained after saturation and stability of these angles, which is shown by the plot of angles versus time graph.

**Table 4:** Test Cases for different applied pressures on different chambers of endoscope.

	Case 1	Case 2	Case 3
Applied Pressure	0.2 bar on Torsion chamber and 0.2 bar on Ventral chamber	0.2 bar on Dorsal chamber and 0.1 bar on Right Lateral chamber	0.2 bar on Right Lateral chamber and 0.2 bar on Dorsal chamber
Bending Angle Visualization			
Module visualization on ANSYS view			
Plotting of angles versus time			

	Case 4	Case 5
Applied Pressure	0.2 bar on Dorsal chamber, 0.1 bar on Right Lateral chamber and 0.1 bar on Left Lateral chamber	0.2 bar on all the 5 chambers
Bending Angle Visualization		
Module visualization on ANSYS view		
Plotting of angles versus time		

### Dataset generation

Moving to the next stage, we generated a dataset with 500 samples. Each sample consists of a random combination of pressures for each chamber of the above shown chambers and the corresponding twisting angle, bending angle, twisting velocity and angular velocity. The most important parameter for our endoscope is the bending angle which we will focus on. We tried generating a dataset with a limited number of samples, five to ten, but this led to underfitting, where the model fails to capture the full range of possible behavior. To use the dataset and take the full advantage of it, we should make sure that we have different samples and valid ones too. The raw dataset was cleaned to remove inactive, repetitive samples or samples having conditions not likely to happen. We removed the samples with all pressures are zero, these cases occur during sensor initialization and can add noise during training. Then we checked if any sample is repeated with the same combination of pressure inputs to discard it. We have also removed any illogical conditions; we only used realistic readings to get an ideal trusted model^17,23^.

### Machine learning algorithms

Although mathematical models can describe the motion of a single-segment pneumatic soft robot under ideal assumptions, their use in realistic conditions is limited due to hyperplastic material behavior. A data-driven approach is implemented to overcome these limitations and give accurate predictions under real operating conditions. Also, this approach is faster than analytical methods to get the corresponding bending angle with changing pressure for complex surgical applications.

The dataset is prepared using a Python script that takes a list of pressures on random chambers locations and outputs the bending angle for each case based on modeling equations. With the dataset prepared, we moved on to training machine learning models capable of predicting the actuator’s bending angles based on the applied pressure inputs. To achieve this, we implemented two commonly used algorithms: ANN and SVM. Both models were selected for their ability to learn complex, nonlinear relationships from data. In the following part of this section, we will explain in detail how each model was developed and trained.

Before moving to the implementation of each algorithm, data preprocessing should be accomplished first to have trusted data. After the filtration of data mentioned in the above section, we are now confident that all the samples used in our dataset are informative and represent real cases of deformation. The cleaned dataset is divided into a feature matrix (X) , representing the input pressures applied to internal chambers, and a target matrix (y), representing the four output deformation states.

8 $${\text{X }} = \, \left[ {{\mathrm{P}}0,{\text{ P1}},{\text{ P2}},{\text{ P3}},{\text{ P4}}} \right]$$

where P0 is the pressure applied at Dorsal chamber, P1 is the pressure applied at Torsion chamber, P2 is the pressure applied at Lateral Right chamber, P3 is the pressure applied at Lateral Left chamber and P4 is the pressure applied at Ventral chamber.

9 $${\mathrm{y}} = [\theta_{{{\mathrm{bend}}}} ,\theta_{{{\mathrm{bend}}}} , \, \varphi_{{{\mathrm{twist}}}} , \, \varphi_{{{\mathrm{twist}}}} ].$$

where θ_bend_ is the bending angle, θ̇_bend_ is the bending speed, , φ_twist_ is the twisting angle and φ̇_twist_ is the twisting speed.

We will study the four outputs, but we will focus on our results on the bending angle, θ_bend_ as the bending angle is the main and most functionally significant mode of deformation in soft pneumatic actuators. It is the main parameter related to control and interaction with soft robotics applications. Min–Max normalization was used to scale all features and target matrices to a predefined range of [0, 1] to improve training performance and numerical stability^18,23^.

10 $${\mathrm{X}}_{{{\mathrm{scaled}}}} = \frac{{{\text{Xreal }} - {\text{ Xmin}}}}{{{\text{Xmax }} - {\text{ Xmin}}}}$$

After that, the dataset was divided in an 80:20 ratio into training and testing datasets. To guarantee interpretability and precise metric computation, scaling parameters were saved during training and subsequently utilized to inversely transform predictions to their original physical units during evaluation^23^. Table 5 shows a detailed part of the dataset generated, showing that between each sample, a case is added to make all pressure inputs in the home case.

**Table 5:** Training Dataset.

Case id	Dorsal	Ventral	Lateral-L	Lateral-R	Torsion
1	3	2	1	14	14
Home	0	0	0	0	0
2	17	8	12	3	1
Home	0	0	0	0	0
3	20	7	5	3	12
Home	0	0	0	0	0
4	4	14	10	19	8
Home	0	0	0	0	0
5	3	3	16	15	3
Home	0	0	0	0	0

In this work, Artificial Neural Network (ANN) was chosen to model the nonlinear relationship between input pressures and bending angles of the novel soft robotic endoscope. ANN was used as it is efficient in capturing complex nonlinear behaviors without explicit mathematical modeling. Unlike the time-consuming FEA simulations, this allows for fast and accurate angle prediction.

### The proposed ANN model

**Fig. 5:**
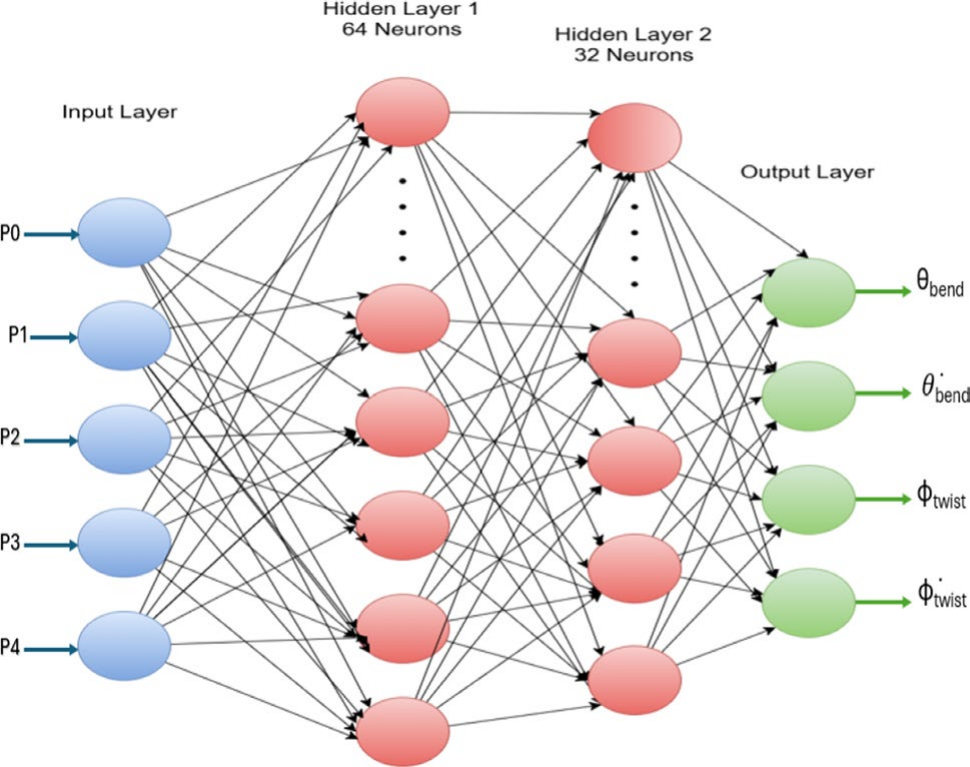
ANN Representation.

A feedforward ANN, Fig. 5, was created using the Multi-layer Perceptron Regressor class from the scikit-learn library to simulate the nonlinear relationship between chamber pressures and the soft actuator’s mechanical response. Five neurons served as the model’s input layer, representing the pressure readings in the five actuator chambers. Two hidden layers, with 64 and 32 neurons each, were then added. Four neurons, representing the target mechanical responses of bending angle, twist angle, bending speed, and twist speed, were present in the output layer.

All hidden layers used the Rectified Linear Unit (ReLU) activation function to add nonlinearity and allow the network to learn complex mappings^17,23^. The ReLU function is defined as:

11 $${\mathrm{f}}\left( {\mathrm{x}} \right) \, = {\text{ max }}\left( {0,{\mathrm{x}}} \right)$$

Due to its computational efficiency, and ability to prevent vanishing gradients, which enhance generalization, this function is commonly used in deep learning. For continuous-valued regression tasks, the output layer employed a linear activation function, which enables the network to generate output over the whole real-valued range without saturation^24^.

Adam (Adaptive Moment Estimation) optimizer has been used in our model, which combines the benefits AdaGrad and RMSProp. Adam adapts the learning rate for each individual weight using estimates of the first and second moments of the gradients, which accelerates convergence and improves stability. The model was trained with a maximum of 1000 iterations and a fixed seed was set to ensure reproducibility, which means ensuring that the results are reproducible every time we run the code^17,24^.

The goal of learning process is to minimize the Mean Squared Error (MSE) between predicted and actual values^24^ defined as:

12 $$MSE=\frac{1}{n}{\sum }_{i=1}^{n}{\left({y}_{i}-\widehat{{y}_{i}}\right)}^{2}$$

where n is the total number of samples, ${y}_{i}$ is the true value and $\widehat{{y}_{i}}$ is the predicted value.

### Support vector machine (SVM)

In addition to the ANN model, SVM regression model was implemented to get a general model that gets the bending angle outputs based on input pressures. The base SVR model from the scikit-learn library was wrapped in a Multioutput Regressor to enable simultaneous prediction of all four target variables: bending angle, twist angle, bending speed, and twist speed^23^. SVM is an effective kernel-based technique that can be used to model nonlinear relationships by converting input data into a higher-dimensional space that allows for the construction of a linear regression function. We used a well-known kernel, Radial Basis Function (RBF), which is known for its flexibility and reliability in capturing non linearities^18,24^. The RBF kernel calculates the similarity between data points based on their Euclidean distance, defined as:

13 $$K\left(x,{x}{\prime}\right)=\mathrm{exp}\left(-\upgamma |x-{x}{\prime}{|}^{2}\right)$$

where γ controls how far the influence of each training example extends. In our implementation, we used the default setting from scikit-learn, where γ = $\frac{1}{n}$ , where n is representing the number of features, which is equal to 5.

The regularization parameter C is set to 10 to create a strong regression model. This number indicates how much the model values maintaining a smooth prediction function over lowering training errors. We also set the epsilon-insensitive loss function with ε = 0.1, which enables the model to only concentrate on larger, more significant deviations and ignore small errors, or those that fall inside the margin of tolerance ^18,23^. SVM works by finding the simplest possible function, f(x), that stays closer to the training data, without exceeding the allowed error margin (ε)^24^. This is done using the following cost function:

14 $$\frac{1}{2}|w{|}^{2}+C{\sum }_{i=1}^{n}\left({\upxi }_{i}+{\upxi }_{i}^{*}\right)$$

## Experimental testing

In this section, we will discuss the fabrication process of the proposed endoscopic design to validate the AI model developed.

### Mold design

Initially, a cylindrical mold with built-in chamber holes was created as a single piece. However, this concept has two primary problems. First, the small size and complicated, uneven internal geometry of the cured silicone endoscope made it difficult to remove the mold. The endoscope was often removed using external sharp tools, which raises the possibility of structural defects. Breaking the mold would have preserved the integrity of the endoscope, but doing so is costly and impracticable, especially when it comes to production. The second issue was the partial curing of the silicone. The enclosed design of the single-piece mold occasionally resulted in uncured portions because it restricted ventilation and extended the curing time, particularly in the deeper internal chambers^25^.

A multi-part mold was developed to solve these problems addressed in the first mold’s design. It is made up of three parts: a mold wall to construct the outer cylinder, a wall ring part to join them, and a base and base T-slot to facilitate mold removal. It also includes the base core, which creates the endoscope’s internal chambers. In order to ensure precise alignment and sealing during casting, these components are made to interlock via an interference fit.

### Mold’s fabrication and module’s casting

**Fig. 6:**
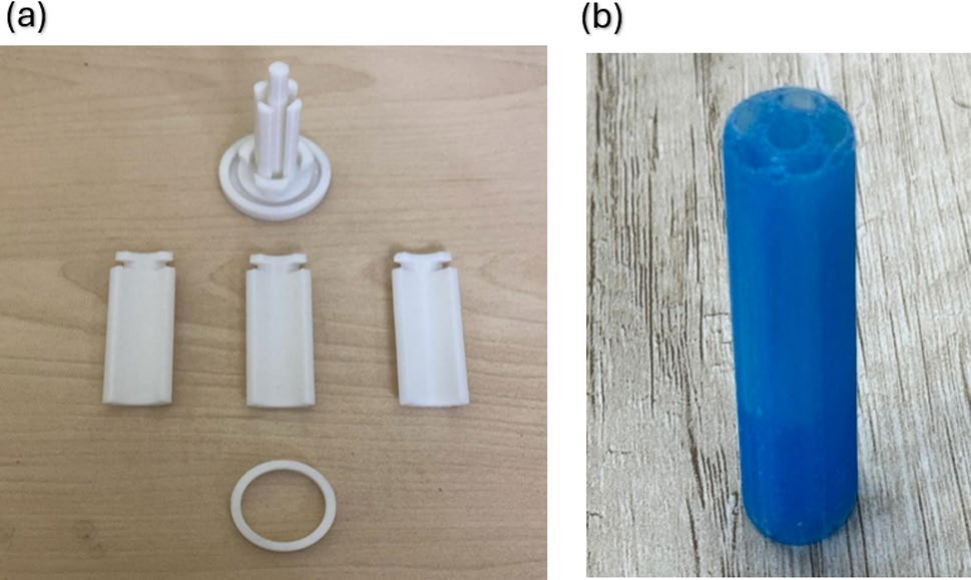
(**a**) Mold’s fabricated parts, (**b**) Physical Prototype of endoscope.

A Fused Deposition Modeling (FDM) 3D printer was used to create the mold using 1.75 mm PLA filament, which was selected for its dimensional stability and suitability for silicone casting at room temperature. Ultimaker Cura was used to slice the design, enabling us to prepare the model for 3D printing with precise and robust settings. The mold, shown in Fig. 6a, was printed with a 0.2 mm layer height, infill of 100%, and a nozzle of 0.4 mm to ensure stifness. The printing process of the mold took approximately 4 h due to the high accuracy achieved.

In casting process, Ecoflex 00–50 is used due to its high flexibility. The silicone was mixed until uniform and then placed in a vacuum chamber to remove trapped air bubbles. After degassing, it was slowly poured into the mold from the top to ensure smooth filling of all chambers. The mold was left at room temperature for 2 h to allow complete curing. Once cured, the mold was carefully opened by hand, and the endoscope was removed without damaging the silicone or the mold. The final physical prototype of the mold is shown in Fig. 6b.

## Results and discussion

In order to illustrate the performance of the proposed design and AI-Model, experimental work has been done using Ansys and Python. The finite element simulations, performed on Ansys software, successfully demonstrated the response of the proposed design due to different applied pressure. The results show how changing applied pressure affects the bending angle of the endoscope. Increasing the pressure in one chamber or applying pressures on opposite chambers produced higher bending angles.

**Fig. 7:**
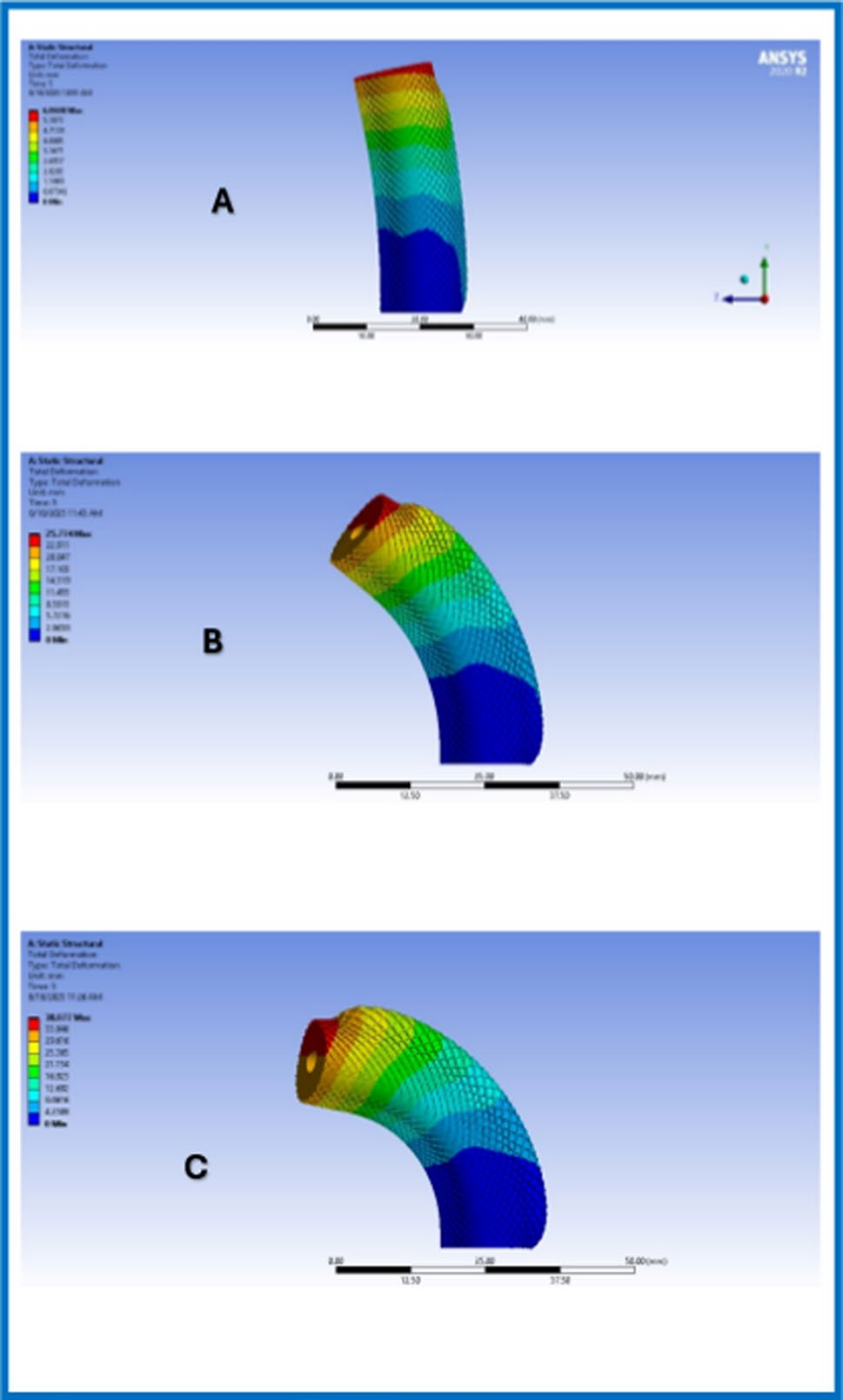
(**A**) Ansys results with pressure 1 bar and bending angle of 92°, (**B**) Ansys results with pressure 1.5 bars and bending angle of 100°, (**C**) Ansys results with pressure 2 bars and bending angle of 120°

These results, Fig. 7, demonstrate that the proposed design is efficient, and the angles obtained are as desired. However, Ansys simulation did not capture the twisting angle which is a very important parameter to measure. Twisting angle may affect the effect of the bending angle. Thus, we developed an AI- model to validate our results and obtain the corresponding twisting angles for each case.

**Fig. 8:**
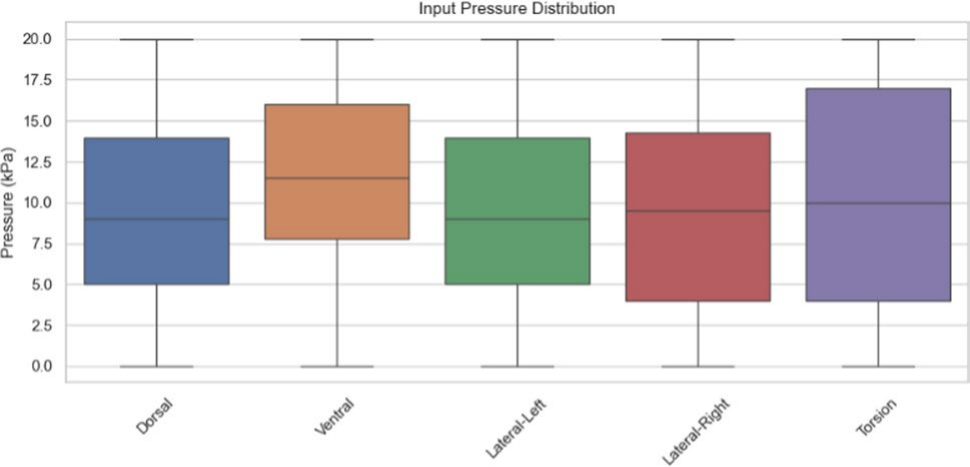
Dataset Input Pressure Distribution.

AI models using ANN and SVM have been developed to validate the proposed design’s capability at different cases of pressure and chambers activation. Figure 8 shows the different input pressure distribution on each chamber, which is the input dataset used to validate our results using Python.

**Fig. 9:**
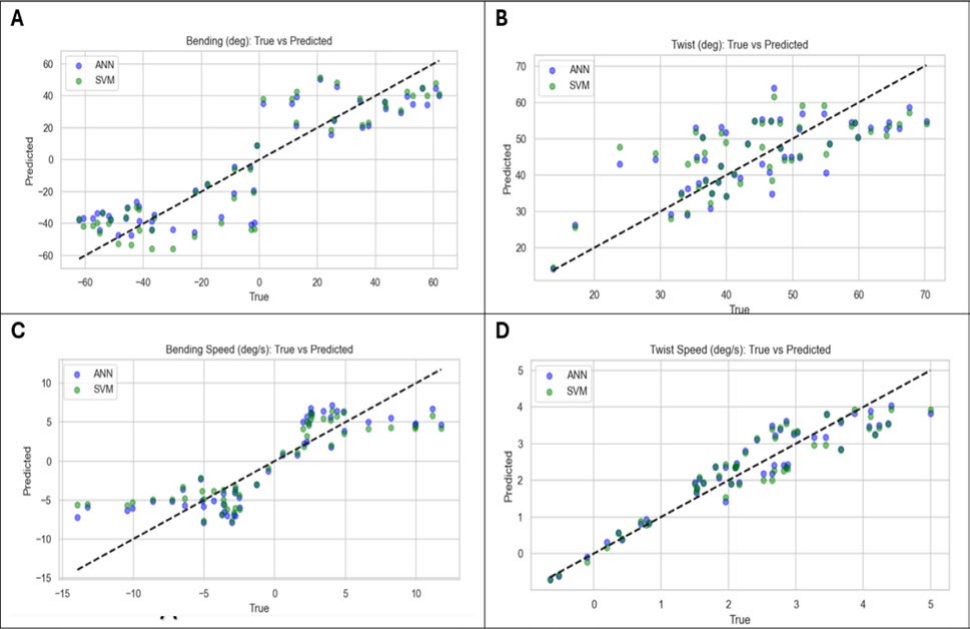
(**A**) Predicted output of ANN and SVM model of bending angle versus time at different cases, (**B**) Predicted output of ANN and SVM model of twisting angle versus time at different cases, (**C**) Predicted output of ANN and SVM model of bending speed versus time at different cases, (**D**) Predicted output of ANN and SVM model of twisting speed versus time at different cases.

**Fig. 10:**
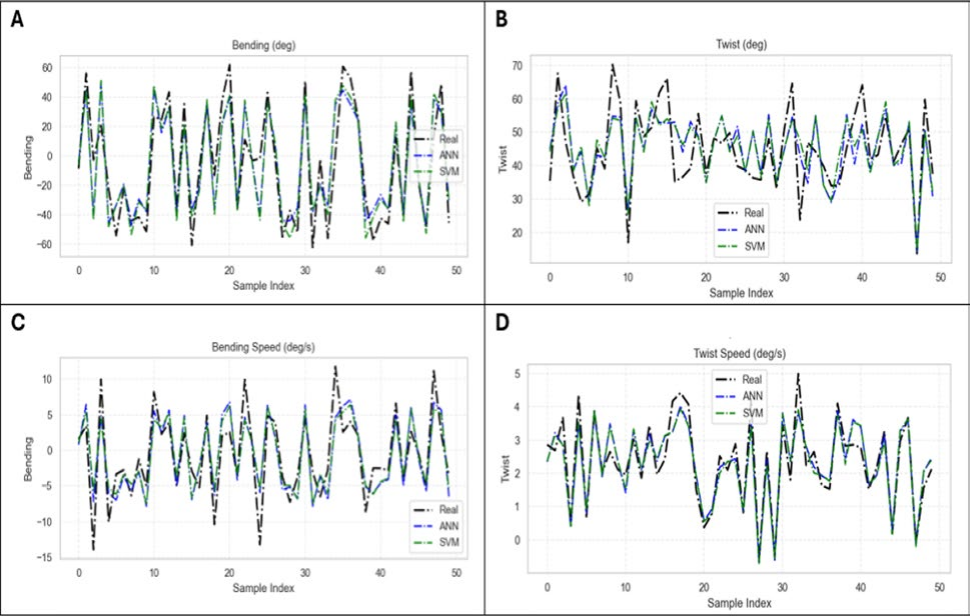
(**A**) Model Output versus FEA results for bending angle using ANN and SVM, (**B**) Model Output versus FEA results for twisting angle using ANN and SVM, (**C**) Model Output versus FEA results for bending speed using ANN and SVM, (**D**) Model Output versus FEA results for twisting speed using ANN and SVM.

Figures 9 and 10 show the results of AI-Models for predicting bending angles of the soft robot endoscope, proving its accuracy in converting chambers pressures to corresponding deformation. Figure 9 illustrates the relationship between true values obtained from FEA model and predicted values obtained from AI model for bending angle, twisting angle, bending speed and twisting speed using ANN and SVM.

Figure 10 illustrates the relationship between the actual performance of the model using testing dataset versus the FEA results, where they are almost close with only minor deviations.

From these results, we can find that the superior performance of ANN in predicting the bending angle. But for the rest of parameters, the two algorithms’ results are nearly accurate.

All the models are developed and compared using the two algorithms implemented. The previous figures show the results for 50 random samples only for each parameter. The comparison of the different models used to predict the bending angle of our novel endoscope design indicates that ML algorithms successfully met the results of FEA model.

**Fig. 11:**
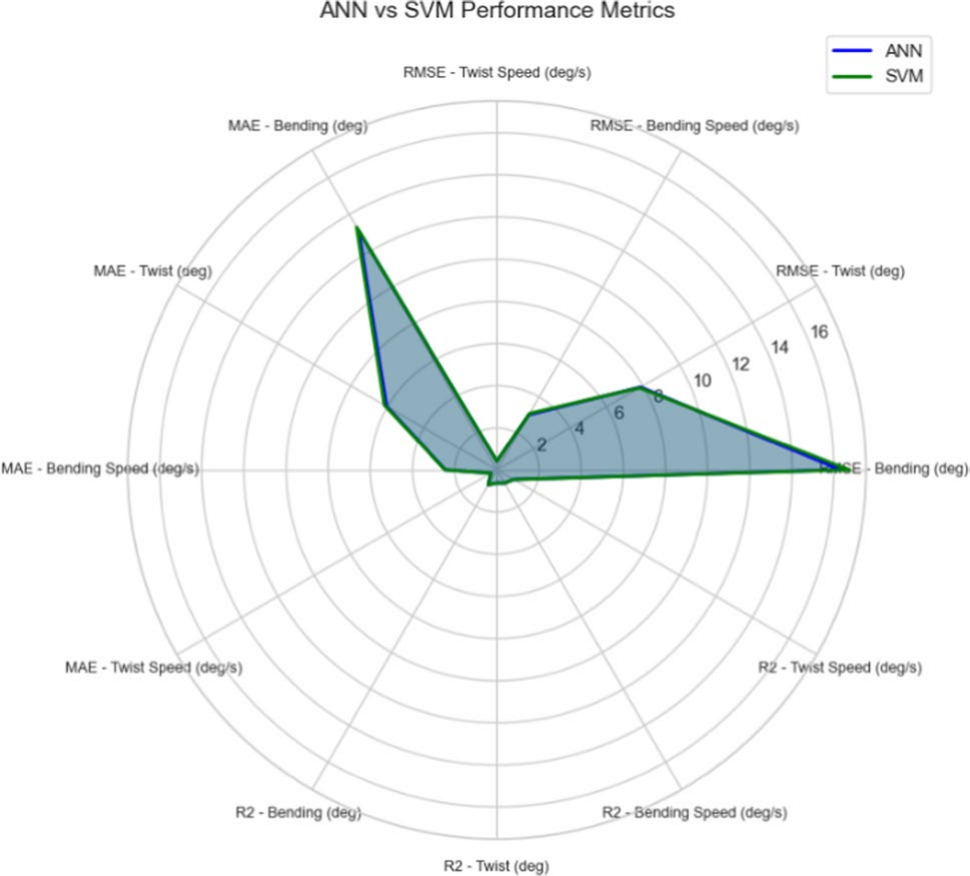
Performance Metrices for ANN and SVM Algorithms.

The performance of the proposed AI-model, Fig. 11, was tested using standard performance metrices: Root Mean Squared Error (RMSE), Mean Absolute Error (MAE) and the coefficient of determination (R^2^). For ANN algorithm: the model achieved a RMSE of 15.18° and a MAE of 11.97°. Regarding R^2^ value, it was recorded to be 0.85. For SVM algorithm, the model reached a RMSE of 15.63°, a MAE of 12.01° and R^2^ of 0.84. These results prove that the AI-Model developed is highly accurate in predicting the bending behavior obtained by Ansys simulation, and also in predicting the twisting angles to study its effect on total deformation.

The proposed AI-based model and FEA model were validated by experimental testing. The numerical model accurately agrees with the actuator’s primary mechanical behavior since the experimentally measured deformation values fall within the same range and exhibit good agreement with the FEA predictions. Material nonlinearity, fabrication tolerances, and measurement error are responsible for small deviations between the simulated and experimental results. Overall, the experimental findings validate the accuracy of the FEA and AI models in predicting the deformation behavior of the actuator.

**Fig. 12:**
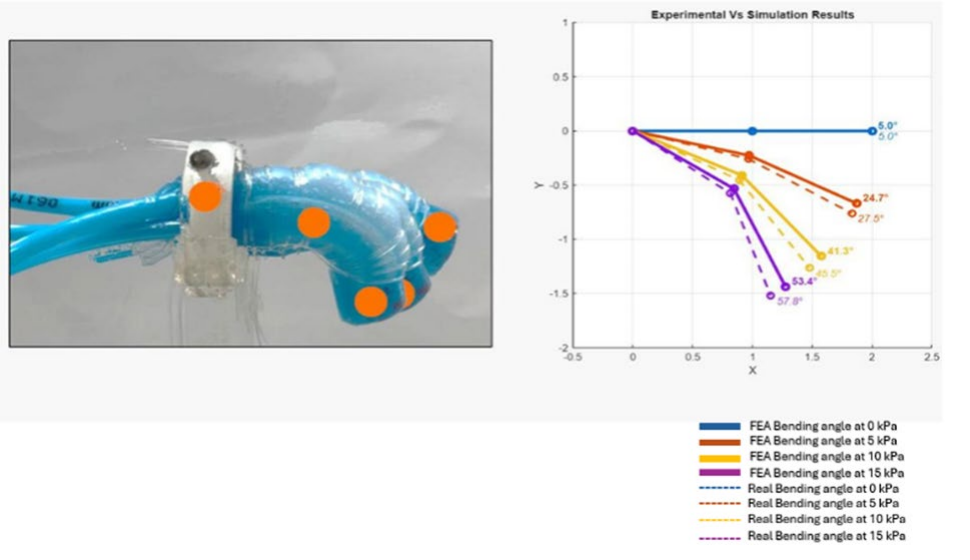
Experimental Results

The performance of the proposed soft endoscope was evaluated by examining the FEA results and testing the physical prototype with real-time bending angle computation. Different pressures were applied to the physical prototype and to the FE model to validate the design. Figure 12 shows the results for different test cases. At pressure 0 kPa, the bending angle for FE model and physical prototype fluctuates between 0° to 5°. At pressure of 5 kPa, simulation’s bending angle obtained was 24.7°, while experimental was 27.5°. At pressure 10 kPa, simulation’s bending angle was found to be 41.3°, while experiment’s bending angle was 45.5°. At pressure of 15 kPa, simulation’s result was 53.4°, while experiment’s result was 57.8°. It is noticed that the bending angle obtained from operating the physical prototype is higher than that obtained from the FE model. This difference is caused due to Yeoh hyperplastic formulation used in FE model which captures continuously the non-linearity in response, which in most cases estimates a larger stiffness at lower pressures. On the other hand, some soft spots may be found in the real physical prototype due to variations in curing and mixing ratios.

To evaluate the repeatability of the proposed soft robotic endoscope, the experiment was repeated 3 times at pressure 15 kPa with the same conditions. The measured bending angles in the three trials were 57.8°, 55.8°, and 58°, respectively. These measurements resulted in a mean bending angle of 57.2° with a standard deviation of 1.22°, indicating very low variation between repeated tests.

## Conclusion and future work

MIS has become an essential approach in modern medicine, offering patients faster recovery, less pain, and less risk compared to traditional surgeries. The effectiveness of MIS mainly depends on the design and capabilities of endoscope in navigating complex anatomical pathways while maintaining patient safety. However, endoscopes face major challenges in reaching higher bending angles to reach complex areas while maintaining minimum pressure.

In this research, a new design is proposed to overcome the previous challenges. The proposed module achieved a bending angle of 90° at a pressure of 0.2 bars reflecting an improvement of over 10% compared to previous designs reported in literature, with better flexibility. The design is fabricated with high accuracy and tested to validate the concept. These enhancements make the new design a good idea for creating safer and more flexible endoscopes that give surgeons better control during minimally invasive procedures, while also improving access to difficult or more critical anatomical areas due to their increased bending capability.

In parallel with the hardware improvements, an artificial intelligence model was developed to predict the bending angle under different input conditions. The model demonstrated high accuracy, making it a valuable tool for rapid performance evaluation and design optimization. By integrating AI with soft robotic technology, the reliance on repeated physical testing is reduced, contributing to the advancement of endoscopes with enhanced adaptability and efficiency. Also, developing an AI model reduces high computational power needed for FEA to try each combination of inputs to find the bending angle. Although twisting angles can be extracted from FEA, the proposed AI model enables fast and accurate prediction of twisting angles for real-time operation. Twisting angle measurement is one of the key parameters as it may affect the final deformation. The analysis of AI-model’s performance proved that ANN algorithm is more accurate and reliable than SVM algorithm in our application.

Future work will concentrate on improving the module’s reliability and performance in actual surgical applications. In order to provide smoother motion in complex pathways, the design could be expanded to multiple segments rather than just one. Closed-loop control should be employed to gain more exact input for the resulting bending angles to correct any non-linearities throughout the module’s navigation. Finally, we can add control strategies to the system to make it safer for surgeries.

## Supplementary Information

Below is the link to the electronic supplementary material.

Supplementary Material 1

## Data Availability

The datasets used and/or analyzed during the current study available from the corresponding author on reasonable request.
