# Supplementary figures and images for: Design of a soft robotic endoscope with enhanced bending and AI-based prediction
Source: Sci Rep. 2026 Apr 20;16:12878. doi: 10.1038/s41598-026-46334-y (PMC13096537; doi:10.1038/s41598-026-46334-y)

## Slide 1
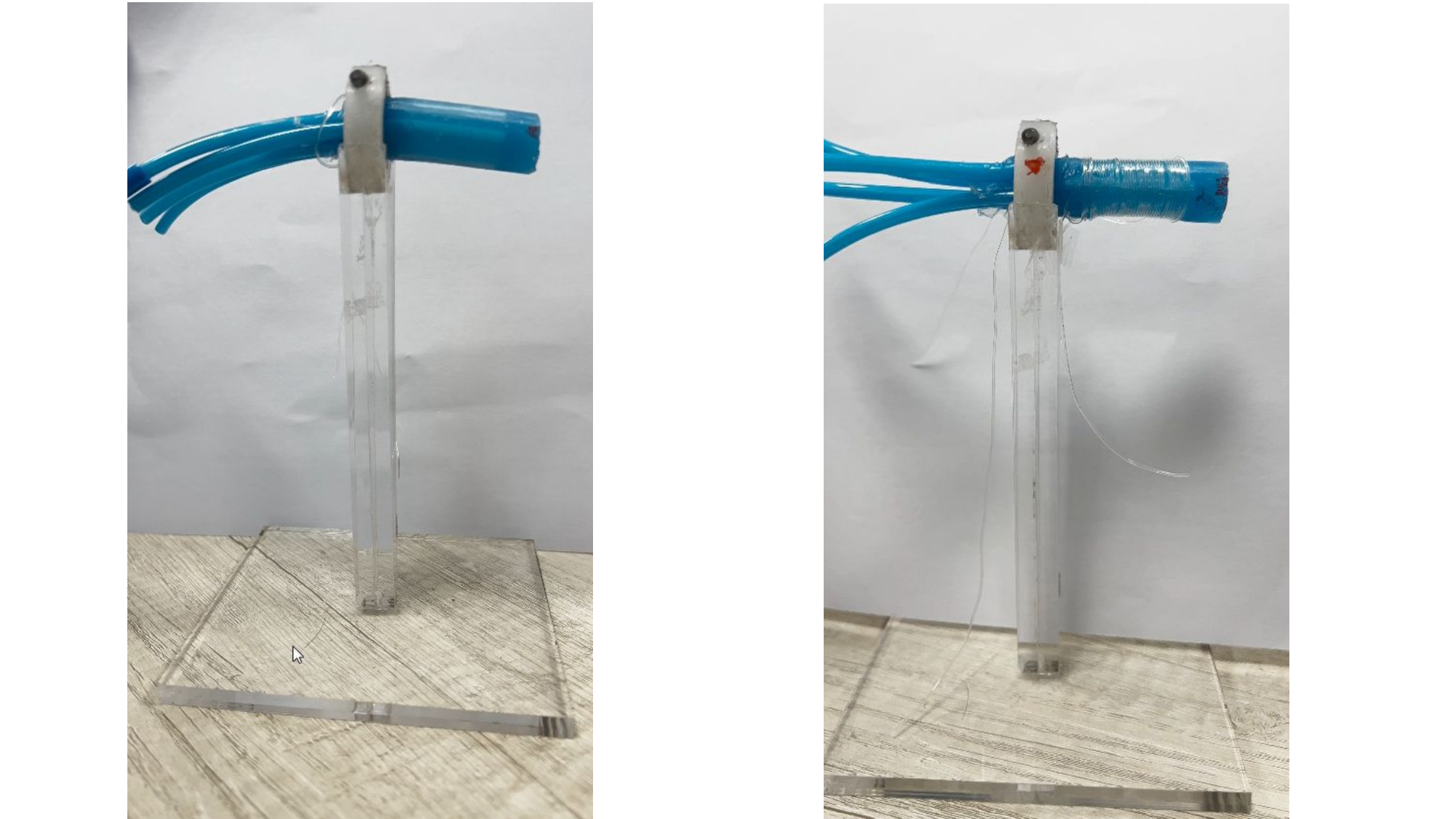

#

Supplement: Supplementary file 1 — Supplementary Material 1 [file 41598_2026_46334_MOESM1_ESM.pptx]
